# The Crosstalk Between Epigenetic Mechanisms and Alternative RNA Processing Regulation

**DOI:** 10.3389/fgene.2020.00998

**Published:** 2020-08-20

**Authors:** Jian Zhang, Yi-Zhe Zhang, Jing Jiang, Cheng-Guo Duan

**Affiliations:** ^1^Shanghai Center for Plant Stress Biology, CAS Center for Excellence in Molecular Plant Sciences, Chinese Academy of Sciences, Shanghai, China; ^2^University of Chinese Academy of Sciences, Beijing, China; ^3^State Key Laboratory of Crop Stress Adaptation and Improvement, School of Life Sciences, Henan University, Kaifeng, China

**Keywords:** RNA processing, alternative splicing, alternative polyadenylation, epigenetics, DNA methylation, histone modifications

## Abstract

As a co-transcriptional process, RNA processing, including alternative splicing and alternative polyadenylation, is crucial for the generation of multiple mRNA isoforms. RNA processing mechanisms are widespread across all higher eukaryotes and play critical roles in cell differentiation, organ development and disease response. Recently, significant progresses have been made in understanding the mechanism of RNA processing. RNA processing is regulated by *trans*-acting factors such as splicing factors, RNA-binding proteins and *cis*-sequences in pre-mRNA, and increasing evidence suggests that epigenetic mechanisms, which are important for the dynamic regulation and state of specific chromatic regions, are also involved in co-transcriptional RNA processing. In contrast, recent studies also suggest that alternative RNA processing also has a feedback regulation on epigenetic mechanisms. In this review, we discuss recent studies and summarize the current knowledge on the epigenetic regulation of alternative RNA processing. In addition, a feedback regulation of RNA processing on epigenetic regulators is also discussed.

## Introduction

Messenger RNA production is a fantastically complex process in eukaryotes, including transcription of mRNA precursors followed by capping, splicing, and polyadenylation. Alternative RNA processing, including splicing and polyadenylation (AS/APA), leads to the formation of distinct mRNA isoforms and explains how massive proteomic complexity can be accomplished with the relatively few genes in higher eukaryotes (Elkon et al., 2013; Tian and Manley, 2016). AS/APA are mechanisms widespread across all eukaryotic species, from yeast to humans and plants. Recent advances based on a vast amount of high-throughput sequencing data indicate that nearly 95% of multi-exon mammalian genes undergo alternative splicing (Pan et al., 2008; Barash et al., 2010) and more than 70% of mammalian genes express APA isoforms (Derti et al., 2012; Hoque et al., 2013). AS/APA have gained renewed and expanded consideration as crucial regulators of gene expression and contribute to development and cellular differentiation and proliferation, neuron activation and other biological processes (Hong et al., 2018; Xu and Zhang, 2018; Fan et al., 2018; Yoshimi et al., 2019).

Traditionally, alternative RNA processing has been thought to be predominantly controlled by both *cis*-regulatory sequences and *trans*-acting factors. In AS regulation, *cis*-regulatory sequences include splicing enhancers and silencers, typically 10 nt in length, the impact of which depends on their location and their preferential splice sites (Cáceres and Kornblihtt, 2002; Cooper et al., 2009). *Trans*-acting factors activate, whereas other factors inhibit, the use of splice sites, by binding to splicing enhancers and silencers (Jelen et al., 2007; Han et al., 2010). Similar to AS, the combined effects of multiple *trans*-acting factors and *cis* elements clearly determine the likelihood of diverse poly(A) site usage (Movassat et al., 2016; Tian and Manley, 2016).

Despite the wide acceptance that these *cis*-regulatory sequences and *trans*-acting factors regulate alternative RNA processing, AS and APA are more complicated processes in co-transcriptional events than originally anticipated. Here, we review the implications of the recently exposed roles of epigenetic mechanisms, such as DNA methylation, histone modifications, histone variants, and some non-coding RNA (ncRNA) in alternative RNA processing regulation. A feedback of alternative RNA processing on epigenetic regulation was also discussed.

## Chromatin-Based Regulation of Alternative RNA Processing

### DNA Methylation and Alternative RNA Processing

DNA methylation, resulting in 5’ methylation of cytosine (5mC), is a conserved and heritable DNA modification that affects gene expression in a genome-wide manner (Li and Zhang, 2014). The impact of DNA methylation on gene expression varies depending on its genomic contexts. The role of promoter DNA methylation in gene expression has been well investigated, which is widely believed to cause transcriptional inhibition of downstream genes (Law and Jacobsen, 2010). Interestingly, recent studies in model plant *Arabidopsis* revealed that two SU(VAR)3–9 homologs, SUVH1 and SUVH3, bind to methylated DNA and recruit the DNAJ proteins to enhance proximal gene expression, thereby counteracting the repressive effects of transposon insertion near genes (Harris et al., 2018; Xiao et al., 2019; Zhao et al., 2019). Compared to DNA methylation in promoter regions, the function of genic DNA methylation remains elusive (Ball et al., 2009). During the last decade, several studies indicate that genic DNA methylation has a positive effect on the expression of associated genes and prevents spurious transcription initiation, and it is present within a number of cancer-related genes and has been regarded as a hallmark of human cancer (Baylin and Jones, 2011; Yang et al., 2014; Neri et al., 2017).

Recent studies reveal a strong correlation between DNA methylation and alternative splicing. Yang et al. (2014) showed that gene body DNA demethylation mediated by DNA methyltransferase inhibitor 5-aza-2′-deoxycytidine results in reduced efficiencies of transcription elongation or splicing. In human cells, Shukla et al. (2011) reported that a DNA-binding protein, called CCCTC-binding factor (CTCF), can promote inclusion of weak upstream exons by mediating local RNA polymerase II pausing. In this case, DNA methylation inhibits CTCF binding to *CD45* exon 5, which enables Pol II to transcribe more rapidly, giving rise to an exon 5 exclusion (Ong and Corces, 2014). More recently, Nanavaty et al. (2020) further revealed that CTCF is a bifunctional regulator which influences both alternative splicing and alternative polyadenylation. Removal of DNA methylation enables CTCF binding and recruitment of the cohesin complex, which in turn form chromatin loops to promote proximal polyadenylation site usage. These works clearly demonstrate that DNA methylation has an important participation in RNA processing regulation. While, limited information is currently available regarding how DNA binding proteins disturb the elongation of Pol II. It reminded us that there maybe are other factors influencing Pol II elongation in CTCF-mediated AS regulation, like the cohesin complex.

Unlike CTCF protein which binds to unmethylated DNA, a growing number of studies have shown that the methyl cytosine-guanine dinucleotide (CpG) binding protein 2 (MeCP2) binds to methylated regions to influence AS. MeCP2 is the earliest reported multifunctional protein that contains both methyl-CpG-binding domains and transcriptional repressor domains (Nan et al., 1997). Acting as a chromatin adaptor, MeCP2 is attracted to 5mC on alternative exons, triggering its interaction with histone deacetylases (HDACs), which modulate alternative splicing (Maunakea et al., 2013). As we delve deeper into the function of MeCP2, it is becoming clear that MeCP2 recruiting splicing factors to regulate mRNA splicing is also a nearly ubiquitous mechanism in animals (Cheng et al., 2017; Wong et al., 2017).

In plants, the available information regarding whether gene body DNA methylation affects AS and the extent of this mediation is currently limited. The first study of DNA methylation-related functions in splicing was reported in maize (Regulski et al., 2013). More recently, the cytosine methyltransferase OsMET1 was found to affect global AS events in rice, in which a total of 6319 more events were identified with the *met1* mutant compared with those associated with the wild-type strain (Wang et al., 2016). However, deeper research combining DNA methylation and AS/APA in plant is lacking. Whether it has the similar regulatory mechanism with mammals needs to be further elucidated.

### Histone Modification-Mediated Regulation of Alternative RNA Processing

Chromatin structure is dominated by nucleosome density and positioning, as well as by histone modifications and DNA methylation (Duan et al., 2018). In contrast to DNA methylation, more than 50 diverse modifications have been identified on histone tails. Different modifications are linked with distinct functions, such as transcriptional activation or inhibition (Henikoff and Shilatifard, 2011). Recent reports indicate that histone modifications are also involved in the regulation of RNA processing. In fact, the involvement of histone modification in regulation of RNA processing was found earlier than DNA methylation (Luco et al., 2010). Similar to DNA methylation, absence of histone marks results in chromatin structure changes, immediately affecting Pol II elongation and alternative RNA processing.

**FIGURE 1:**
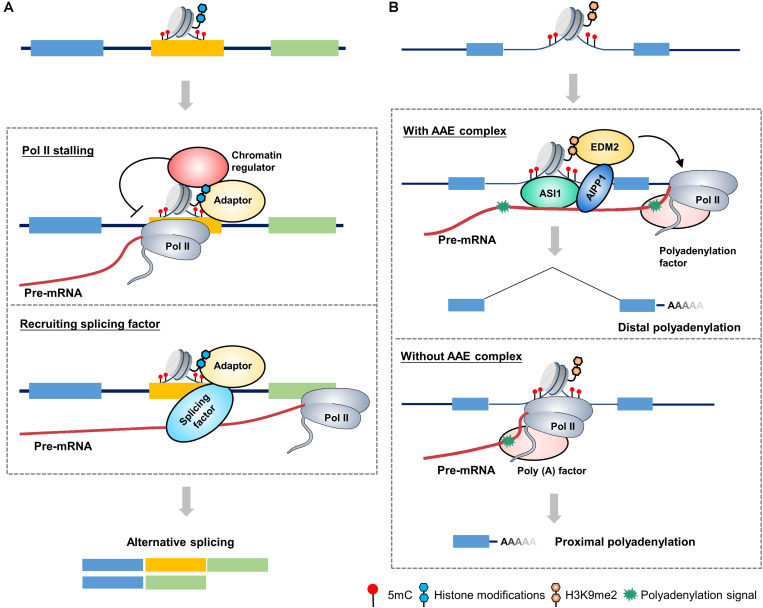
A proposed model for chromatin-based epigenetic regulation of alternative RNA processing. **(A)** A proposed model of chromatin-based regulation of alternative splicing in mammals. Adaptor proteins recognizes and binds to alternative exon, which is marked by epigenetic marks (such as 5mC and histone modifications), to affect alternative splicing through two possible mechanisms: (1) Adaptor protein recruits chromatin regulators (such as chromatin remodelers, cohesion complex, etc.) to change the chromatin status of alternative exon, leading to a stalling of Pol II elongation, which in turn favors the retention of alternative exon. (2) Adaptor protein directly recruits splicing-related factors to promote the retention of alternative exon. **(B)** A proposed model of chromatin-based regulation of alternative polyadenylation in plants. The ASI1-AIPP1-EDM2 (AAE) complex recognizes and binds to the intronic heterochromatin elements (such as 5mC and H3K9me2) and corresponding pre-mRNA, favoring the passthrough of elongating Pol II, thereby promoting the usage of distal polyadenylation signal. When the AAE complex is absent, Pol II elongation is slowed down at intronic heterochromatin region, which favors the usage of proximal polyadenylation signal. Different colored boxes in **(A)** and **(B)** represent exons.

Histone H3 lysine 36 trimethylation (H3K36me3) mark is an active mark and is abundant in actively transcribed gene bodies (Liu et al., 2010). It has been shown that dysfunction of SETD2, an H3K36me3 methyltransferase, induced changes in 186 AS events (Yuan et al., 2017). In humans, the MORF-related gene on chromosome 15 (MRG15) is a well-established model system to study the interplay between histone modifications and the splicing machinery. The H3K36me3 mark influences splicing by impacting the recruitment of splicing regulators through a chromatin-binding protein, that is, MRG15. In this mechanism, the H3K36me3 mark serves as anchors for MRG15 binding, which in turn recruits the splicing regulator polypyrimidine tract-binding (PTB) to pre-mRNA (Figure 1A). The H3K36me3–MRG15–PTB complex forms a chromatin-splicing adaptor system regulating numerous splicing events, including FGFR2 splicing, which is essential for tumor growth and invasion of lung cancer (Sanidas et al., 2014; Naftelberg et al., 2015).

In contrast to the H3K36me3–MRG15–PTB complex which favors exclusion of alternative exons, diverse histone modifications can lead to a diametrically opposite splicing pattern. Heterochromatin protein 1 (HP1), which has three isoforms in humans, HP1α, HP1β, and HP1γ, binds directly to histone H3 lysine 9 trimethylation (H3K9me3; Bannister et al., 2001). A previous study indicated that HP1γ forms an additional link with chromatin, binding to the coding region where it associates with pre-mRNA and favoring its transient retention on chromatin. The modification to the chromatin structures of the *CD44* gene slows the elongation rate of Pol II, which in turn facilitates the recruitment of splicing factors such as U2AF65 and PRP8 to alternative exons, resulting in the inclusion of alternative exons (Saint-André et al., 2011; Yearim et al., 2015). Unsurprisingly, diverse adaptor proteins at H3K9me3 lead to distinct splicing patterns. HP1α and HP1β bind to methylated alternative exons and recruit the splicing factor serine/arginine-rich splicing factor 3 (SRSF3), thus enhancing the role of as a splicing silencer and reducing the number of induced alternative exons (Yearim et al., 2015).

In plants, *Arabidopsis* encodes two homologs of human MRG15, MRG1 and MRG2, which bind to H3K4me3/H3K36m3-modifying histone marks and trigger temperature-induced flowering via the florigen gene *FT* (Bu et al., 2014). However, it seems like that MRG1/2 have diversified from their animal homologs during evolution, yet they still maintain their conserved H3K36me3-binding molecular function (Xu et al., 2014; An et al., 2020; Guo et al., 2020). Recently, a protein complex in *Arabidopsis*, called anti-silencing 1 (ASI1)-ASI1 immunoprecipitated protein 1 (AIPP1)-enhanced downy mildew 2 (EDM2) (AAE) complex, was identified targeting genic heterochromatic elements to regulate APA (Duan et al., 2017). In this complex, ASI1, also named IBM2 and SG1 (Saze et al., 2013; Coustham et al., 2014), is a plant-specific chromatin regulator which bears chromatin- and RNA-binding capacity through its bromo-adjacent homology (BAH) and RNA recognition motif (RRM) domains, respectively (Wang et al., 2013). EDM2 is a multifunctional chromatin regulator containing two and half plant homeodomains (PHDs). Its PHD fingers have the binding capacity of H3K9me2 and other histone modifications (Lei et al., 2014). ASI1 and EDM2 associate *in vivo* through an RRM motif-containing bridge protein AIPP1 (also named EDM3; Duan et al., 2017). The AAE complex can bind to intronic heterochromatin, most of which come from insertions of epigenetically silenced transposable and repetitive elements, promoting the usage of distal polyadenylation site (Figure 1B). Dysfunctions of the AAE complex lead to ectopic accumulations of proximally polyadenylated short transcripts. Thus, the AAE complex is indispensable for the generation of full-length transcripts of genic heterochromatin-containing genes. Regarding the underlying mechanism, recent report indicates that EDM2 and AIPP1 mutations can slow down Pol II elongation rate at proximal polyadenylation site, leading to a promotion of proximal polyadenylation site usage (Lai et al., 2019). AAE complex-mediated polyadenylation regulation plays an important role in multiple biological processes, including modulating plant immunity by targeting innate immunity receptor gene *RPP7* (Tsuchiya and Eulgem, 2013), epigenome regulation by targeting histone H3K9me2 demethylase gene *IBM1*, and T-DNA suppression (Saze et al., 2013; Wang et al., 2013). Similar mechanism may also exist in other plants, like bamboo and oil palm (Wang et al., 2017). For example, in oil palm, loss of *Karma* transposon methylation leads to ectopic splicing of the homeotic gene *DEFICIENS*, which accounts for the mantled soma clonal variant phenotype of oil palm (Ong-Abdullah et al., 2015). Interestingly, recent study indicates that FPA, a flowering time regulator in *Arabidopsis*, can antagonize ASI1 in the selection of polyadenylation site. In the double mutant of *asi1* and *fpa*, the polyadenylation pattern phenocopies *fpa* but not *asi1*. While, this antagonistic control only occurs in specific target genes, indicating a complex regulation of AAE complex-mediated polyadenylation (Deremetz et al., 2019).

### Histone Variants and Chromatin-Remodeling Factors

Nucleosome, consisting of 147-bp double-stranded DNA and a single histone octamer, is the basic unit of chromatin. Histone variants, which are transcribed from separate genes, have been shown playing key roles in the regulation of chromatin features. This finding reminds us that histone variants may also regulate co-transcriptional RNA processing. In mammals, five somatic H1 variants (H1.1 to H1.5) have been identified (Happel and Doenecke, 2009). More recently, Glaich et al. (2019) reported that H1.5 deposition is observed at the splicing sites of the short exons in human lung fibroblasts (IMR90 cells), and Pol II on H1.5-marked exons exhibits greater stalling than it does on unmarked exons. Deletion of H1.5 affects the inclusion of short exons with relatively long introns and reduces Pol II occupancy on these exons (Glaich et al., 2019). This finding clearly indicates that the linker histones participate in the regulation of alternative RNA processing, which has not been previously demonstrated (Glaich et al., 2019).

In addition to histone variants, chromatin remodeling factors also affect chromosome segregation and transcription (Clapier and Cairns, 2009). During the last two decades, a growing number of studies have indicated that chromatin remodeling factors also play a role in alternative splicing. Brahma (BRM), the core adenosine triphosphatase (ATPase) subunit of the switch/sucrose nonfermenting (SWI/SNF) chromatin-remodeling complex, was firstly shown to facilitate the inclusion of alternative exons by interacting with Pol II to induce its stalling (Figure 1A; Batsché et al., 2006; Jancewicz et al., 2019). Actually, chromatin remodeler mediated-regulation of AS is an evolutionarily conserved mechanism across most species, such as in maize. ZmCHB101, a SWI3D protein, has been shown controlling AS by altering chromatin status and transcriptional elongation rates under osmotic stress (Yu et al., 2019), although the mechanism by which chromatin remodeling factors interact with Pol II transcription to impact mRNA processing machinery remains unclear.

## Non-Coding RNAs and Alternative RNA Processing

In addition to the identification of many alternative RNA processing events based on chromatin level, an interesting finding suggests that ncRNAs may play a key role in RNA processing regulation (Kishore and Stamm, 2006). Generally, ncRNAs are divided into two groups according to their size: small ncRNAs (< 200 bp), including rRNA, microRNA (miRNA), small nuclear RNA (snRNA), small nucleolar RNA (snoRNA), small interfering RNA (siRNA), and piwi interacting RNA (piRNA); long ncRNAs (> 200 bp, lncRNA; Bartel, 2009). ncRNAs are now commonly believed to have a variety of biological functions, and it is possible that certain ncRNAs catalyze some steps of the splicing reaction (Cech and Steitz, 2014).

### snoRNAs

**FIGURE 2:**
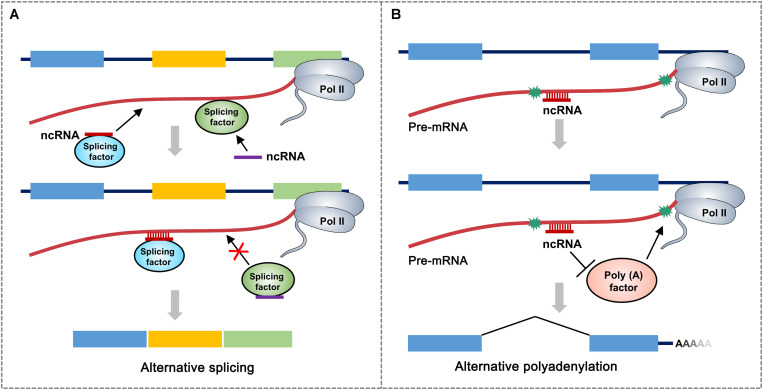
A proposed model for non-coding RNA (ncRNA)-mediated regulation of alternative RNA processing. **(A)** ncRNA directly interacts with different splicing factor to influence alternative RNA processing through two possible mechanisms: ncRNA-splicing factor complex recognizes and binds to the junction region of intron and alternative exon to promote the retention of alternative exon. ncRNA can also inhibit the targeting of splicing factor to the splicing site of pre-mRNA. Asterisks represent polyadenylation signals. Rectangular boxes represent exons. **(B)** ncRNA recognizes and binds to polyadenylation signal-flanking sequence of pre-mRNA, which prevents the accession of polyadenylation-related factors, thereby leading to the usage of distal polyadenylation signal. Different colored boxes in **(A)** and **(B)** represent exons.

It is assumed that most snoRNAs, nearly 70 nt in length, are derived from excised introns through exonucleolytic processing (Watkins and Bohnsack, 2012). There are hundreds of different snoRNAs in vertebrates and have even been found in archaea (Terns and Terns, 2002). The first evidence of the participation of snoRNA in AS was snoRNA HBII-52, which regulates the serotonin receptor 2C and is associated with the congenital disease Prader–Willi syndrome (PWS). HBII-52 regulates AS of 5-HT_2C_R by binding to a silencing element in exon Vb. PWS patients do not express HBII-52. They have different 5-HT_2C_R messenger RNA (mRNA) isoforms than healthy individuals (Kishore and Stamm, 2006). Recently, a class of intronic lncRNAs named snoRNA-related lncRNAs (sno-lncRNAs) was identified in humans. The sno-lncRNAs generated from the PWS region associate strongly with Fox family splicing regulators, altering serotonin receptor 5-HT_2C_R splicing (Figure 2A). In patients with PWS, the expression of some specific sno-lncRNAs is downregulated. As a result, these patients have different 5-HT_2C_R mRNA isoforms than healthy individuals, which have been identified during early embryonic development and adulthood (Yin et al., 2012).

Almost all eukaryotic pre-mRNAs and many ncRNAs are subject to cleavage/polyadenylation at the 3′ end, which takes place in macromolecular machinery called the mRNA 3′-processing complex (Tian and Manley, 2016). It has been shown that snoRNAs, which are classified as *trans*-acting RNAs, directly interact with Fip1, a component of the cleavage and polyadenylation specificity factor (CPSF) complex. Small Nucleolar RNA C.D Box 50A (SNORD50A), a U/A-rich C/D-box snoRNA, inhibits mRNA 3 processing by disturbing the Fip1-poly(A) site (PAS) interaction (Figure 2B). SNORD50A depletion leads to more frequent binding of Fip1 to PAS and increases the 3′ processing of target mRNAs containing U-rich sequences (Huang et al., 2017; Shi et al., 2017). Taken together, these studies strongly suggest that snoRNA is an important regulator of polyadenylation for specific genes by serving as an antagonistic RNA. An important question remains for future studies to address: how do ncRNAs bind to neighboring sequences and regulate the interactions between the core mRNA processing factors and processing sites?

### lncRNAs

Recently, lncRNAs have received increasing attention. In human, Metastasis-associated lung adenocarcinoma transcript 1 (Malat1) is the most widely studied lncRNA. Malat1 was first identified in human non-small cell lung cancer (NSCLC; Ji et al., 2003). A number of serine/arginine-rich (SR) proteins, including SRSF1, SRSF2, and SRSF3, associate with Malat1. Absence of Malat1 affects the localization of some splicing factors in the HeLa cell line and leads to changes AS pattern (Blencowe, 2006). However, the loss of Malat1 in normal mice rarely causes global changes in splicing factor levels and results only in the dysregulation of small mRNAs (Zhang et al., 2012). Meanwhile, deletion of Malat1 in mammary carcinoma mice leads to many AS events in genes essential for cell differentiation and tumorigenesis (Arun et al., 2016). It can therefore be proposed that Malat1 regulates AS in specific cells and tissues under particular conditions. In human cells, the lncRNA Gomafu, which is dynamically regulated by neuronal activation, directly binds to the splicing factors QKI and SRSF1 and inhibits their association with the schizophrenia disease-related gene transcripts, thereby affecting alternative splicing (Barry et al., 2014). In *Arabidopsis*, an lncRNA called alternative splicing competitor (ASCO) binds to the highly conserved spliceosome component PRP8a, thereby impairing the recognition of specific flagellin-related transcripts by PRP8a (Rigo et al., 2020). Actually, it has been shown that ASCO can binds to multiple splicing factors, indicating that lncRNAs may integrate a dynamic network to modulate transcriptome reprogramming, including alternative splicing.

In addition to the evidence we discussed above, some ncRNAs are directly or indirectly involved in RNA processing. It has been shown that piRNAs and piRNA biogenesis components affect mRNA splicing of P-transposable element transcripts *in vivo*, resulting in the production of a non-transposase-encoding mature mRNA isoform in *Drosophila* germ cells (Teixeira et al., 2017). In plants, there is a special family of ncRNAs that can confer *de novo* DNA methylation through the RNA-directed DNA methylation (RdDM) pathway, and thereby inducing global AS/APA events (Matzke and Mosher, 2014; Wang and Chekanova, 2016). As the non-coding transcriptome, ncRNAs are important components of the eukaryotic genome. There may be a large number of mechanisms by which ncRNAs enhance the plasticity of the proteome by interacting with mRNA-processing machinery. A deep understanding of this mechanism will open up broad prospects for gene therapy of various diseases, including cancer, and the application of biotechnology in agricultural and human health fields.

## Epigenetic Regulation and Alternative RNA Processing-Mediated Stress Response in Plants

Unlike animals, plants display a high degree of plasticity during growth and development. In plants, to overcome the constant challenge from a rapidly changing environment, specific adaptation mechanisms have been evolved, among which alternative RNA processing is an important strategy (Chaudhary et al., 2019). Recent work has indicated that the role of epigenetic modifications in regulating AS/APA under stress is emerging (Jabre et al., 2019). Temperature is one of the environmental signals that strongly affects plant development. An recent study indicated that temperature variation is memorized by chromatin via H3K36me3 modification, resulting in a specific splicing pattern, which enables a feasible adaptation to stress conditions (Pajoro et al., 2017). Another study showed that genes which are quickly activated under cold stress and differentially expressed at the splicing level, were found to be modified by H3K27me3 in non-stress conditions (Vyse et al., 2020). These reports suggest a dynamic regulation of temperature stress-responsive genes by alternative RNA processing and histone modification. In *Arabidopsis*, the Nuclear speckle RNA binding proteins (NSRs) have been known as regulators of AS functioning in auxin-associated developmental processes such as lateral root formation (Bazin et al., 2018). These proteins were shown to interact with specific alternatively spliced mRNA targets and at least with one structured lncRNA named ASCO (Bardou et al., 2014). The specific interaction of NSR with the ASCO is able to modulate AS patterns of a subset of NSR target genes, thereby impacting auxin response (Bazin et al., 2018). In other plants, specific association between epigenetic regulators and RNA processing factors under stress conditions has also been found. A maize SWI3D protein, ZmCHB101, has been found to impact alternative splicing contexts of a subset of osmotic stress-responsive genes on genome-wide level (Yu et al., 2019). In turn, alternative RNA processing of pivotal regulatory genes confers plants quick response to the changing climate conditions through alteration of reversible epigenetic marks. While, most of the current researches only focus on one aspect of how plants respond to changeable environment. That means, alternative RNA processing impacts the transcriptome of responsive genes or environment change leads to dynamic alterations of diverse epigenetic modifications (Rataj and Simpson, 2014; Calixto et al., 2018; Li et al., 2018). The mechanistic insights into the detailed interplay between epigenetic regulation and AS/APA in changing environment remains largely limited. In addition, the complicated regulatory mechanisms controlling mRNA isoform ratios in a tissue- or condition-specific manner still remain unclear.

## Feedback Regulation of RNA Processiong on Epigenetic Mechanisms

On the one hand, the evidence above supports a notion that chromatin- and ncRNA-based epigenetic mechanisms have a huge impact on the patterns of alternative RNA processing. On the other hand, alteration of RNA processing pattern can also exert an important influence on epigenetic regulation pathways. In agreement with the notion that the majority of protein-coding genes show alternative processing (Elkon et al., 2013; Naftelberg et al., 2015), a number of epigenetic modifier-encoding genes are subjected to RNA processing regulation. As mentioned above, one classic feedback case is *IBM1*, a major H3K9me2 demethylase-encoding gene in *Arabidopsis. IBM1* is a target of the AAE complex which binds to its intronic repetitive sequence region to promote the generation of functional full-length transcript (Saze et al., 2008; Wang et al., 2013). In one aspect, epigenetic regulators required for the formation of intronic heterochromatin facilitates AAE complex targeting. In line with this notion, mutations of DNA methyltransferases MET1, CMT3 and histone H3K9me2 methyltransferase KYP (SUVH4) phenocopy the phenotype observed in the *aae* mutants, resulting in great reduction of functional *IBM1* transcript (Rigal et al., 2012; Duan et al., 2017). In another aspect, reduced expression of IBM1 protein causes an increase of genome-wide H3K9me2 level, which in turn causes genic CHG hypermethylation through recruiting more CMT3 DNA methyltransferase (Duan et al., 2017). Thus, IBM1-AAE interaction implies an interdependency between epigenetic regulation and alternative polyadenylation. Intriguingly, DNA and H3K9me2 methylation levels in IBM1 intronic heterochromatin region were not obviously changed by the dysfunction of the AAE complex (Duan et al., 2017). One possible explanation is that the AAE complex may has a direct participation in the regulation of the epigenetic status of intronic heterochromatin.

Another example is the BAF complex, including at-rich interactive domain-containing protein 1A (ARID1A), which is an evolutionarily conserved chromatin-remodeling factor (Narayanan et al., 2015). A recent study indicated that EWS–friend leukemia integration 1 (FLI1), a well-established ES oncoprotein, plays a precise role in chromatin regulation by interacting with the BAF complex (Boulay et al., 2017). In addition to modulating chromatin organization, EWS–FLI1 also alters the splicing of many mRNA isoforms (Selvanathan et al., 2015). Surprisingly, EWS–FLI1 leads to preferential splicing of ARID1A-L, promoting ES growth, and ARID1A-L reciprocally facilitates EWS–FLI1 protein stability to maintain the expression of ARIDIA-L. The ARID1A-L isoform is essential for the splicing event, and a reduction in both ARID1A isoforms leads to EWS–FLI1 degradation and cell death. The loss of ARID1A-L has been demonstrated as an explanation of its ability to stabilize EWS–FLI1 (Selvanathan et al., 2019). In this EWS–FLI1-ARIDIA system, chromatin remodeling and alternative splicing are both indispensable. Future efforts should be directed at finding interacting components of epigenetic regulation and AS/APA.

In addition, alternative RNA processing events can also lead to the formation of ncRNAs (Memczak et al., 2013). More recently, Ma et al. reported microRNA-mediated phased small interfering RNA (phasiRNA) generation from long non-coding genes coupled with alternative splicing/polyadenylation in litchi (Ma et al., 2018). An miR482/2118-targeted locus generates four primary transcript isoforms through AS/APA, and diverse phasiRNAs generated from these isoforms appeared to target long terminal repeat (LTR) retrotransposons and other unrelated genes. This study raised the intriguing possibility of cross talk between ncRNAs and AS/APA components. In addition, the diverse alternative mRNA processing-mediated protein variants thus generated immediately affect the properties of proteins, resulting in dysfunction of epigenetic regulators, including chromatin modification enzymes and remodeling factors (Lei et al., 2014; Rusconi et al., 2017; Jancewicz et al., 2019).

## Conclusion

Epigenetic modifications are dynamically regulated by different catalytic enzymes and reader proteins. This feature makes epigenetic mechanisms suitable for multiple biological processes, ranging from cell differentiation, development and environmental stress responses. RNA processing, a widespread mechanism of gene expression in eukaryotic cells, also play vital roles in multiple biological processes. During the last two decades, a great deal of efforts has been made in the crosstalk between epigenetic mechanisms and alternative RNA processing. As shown in Figure 1, chromatin modification, such as DNA methylation and histone modifications can inhibit or reinforce the binding of diverse adaptors. These chromatin adaptors induce alternative RNA processing through changing chromatin structure by collaborating with certain chromatin remodelers or the cohesion complex, or directly recruiting RNA processing factors to distinct splicing/polyadenylation site. Most of the current researches have focused on chromatin-based global changes of alternative RNA processing. In fact, it’s a precise mechanism that is dynamically regulated under diverse conditions, such as during development and environmental stresses.

Different from the chromatin-based alternative RNA processing, ncRNA impact AS/APA on RNA level, mainly by disturbing the binding of RNA processing factors (Figure 2). They can bind to splicing/polyadenylation sites and inhibit the targeting of other RNA binding protein. Study on ncRNA-mediated regulation of alternative RNA processing is a promising field, particularly in the field of pharmaceutical research including RNA interference drugs. It may be a very effective method to treat many human diseases, which are caused by inaccurate splicing or polyadenylation, by covering false splicing/polyadenylation site. Therefore, it is important to find more cases of ncRNA-mediated regulation of RNA processing. In addition, deciphering the physiological relevance of the crosstalk between epigenetic regulation and alternative processing is also important toward understanding normal tissue homeostasis and transition to disease.

Study on the interplay between epigenetic regulation and alternative RNA processing is a novel field which is still at an early stage. In addition to the important researches described above, there are still some outstanding questions regarding the underlying mechanism of alternative RNA processing due to the space constraints not discussed in this review, such as the identification of conserved factors involved in such regulation, a comparison of epigenetic regulation in RNA processing between animals and plants, and the precise epigenetic mechanisms of tissue- and environment-specific AS/APA events. Addressing the remaining questions will undoubtedly expand our understanding of the chromatin codes in the regulation alternative RNA processing.

## Author Contributions

JZ and C-GD designed this review. JZ and Y-ZZ wrote the draft. JJ and C-GD edited it. All authors contributed to the article and approved the submitted version.

## Conflict of Interest

The authors declare that the research was conducted in the absence of any commercial or financial relationships that could be construed as a potential conflict of interest.
